# Pulmonary vein narrowing after pulsed field versus thermal ablation

**DOI:** 10.1093/europace/euae038

**Published:** 2024-02-02

**Authors:** Moussa Mansour, Edward P Gerstenfeld, Chinmay Patel, Andrea Natale, William Whang, Frank A Cuoco, Stavros E Mountantonakis, Douglas N Gibson, John D Harding, Scott K Holland, Anitha B Achyutha, Christopher W Schneider, Andrew S Mugglin, Elizabeth M Albrecht, Kenneth M Stein, John W Lehmann, Vivek Y Reddy

**Affiliations:** Massachusetts General Hospital, GRB 815, 55 Fruit Street, Boston, MA, USA; Section of Cardiac Electrophysiology, University of California San Francisco, San Francisco, CA, USA; Heart and Vascular Institute, University of Pittsburgh Medical Center Pinnacle, Harrisburg, PA, USA; Texas Cardiac Arrhythmia Institute, St. David’s Medical Center, Austin, TX, USA; Case Western Reserve University, Cleveland, OH, USA; Helmsley Electrophysiology Center, Mount Sinai Fuster Heart Hospital, New York, NY, USA; Trident Medical Center, Charleston, SC, USA; Lenox Hill Hospital, Northwell Health, New York City, NY, USA; Scripps Clinic and Prebys Cardiovascular Institute, San Diego, CA, USA; Doylestown Hospital, Doylestown, PA, USA; Medpace Core Laboratories, Cincinnati, OH, USA; Department of Electrophysiology, Boston Scientific Corporation, Menlo Park, CA, USA; Department of Electrophysiology, Boston Scientific Corporation, Menlo Park, CA, USA; Paradigm Biostatistics LLC, Anoka, MN, USA; Department of Electrophysiology, Boston Scientific Corporation, St. Paul, MN, USA; Department of Electrophysiology, Boston Scientific Corporation, St. Paul, MN, USA; Lehmann Consulting, Naples, FL, USA; Helmsley Electrophysiology Center, Mount Sinai Fuster Heart Hospital, New York, NY, USA

**Keywords:** Atrial fibrillation, Pulsed field ablation, Pulmonary vein stenosis, Randomized controlled trial

## Abstract

**Aims:**

When it occurs, pulmonary vein (PV) stenosis after atrial fibrillation (AF) ablation is associated with significant morbidity. Even mild-to-moderate PV narrowing may have long-term implications. Unlike thermal ablation energies, such as radiofrequency (RF) or cryothermy, pulsed field ablation (PFA) is a non-thermal modality associated with less fibrotic proliferation. Herein, we compared the effects of PFA vs. thermal ablation on PV narrowing after AF ablation.

**Methods and results:**

*ADVENT* was a multi-centre, randomized, single-blind study comparing PFA (pentaspline catheter) with thermal ablation—force-sensing RF or cryoballoon (CB)—to treat drug-refractory paroxysmal AF. Pulmonary vein diameter and aggregate cross-sectional area were obtained by baseline and 3-month imaging. The pre-specified, formally tested, secondary safety endpoint compared a measure of PV narrowing between PFA vs. thermal groups, with superiority defined by posterior probability > 0.975. Among subjects randomized to PFA (*n* = 305) or thermal ablation (*n* = 302), 259 PFA and 255 thermal ablation (137 RF and 118 CB) subjects had complete baseline and 3-month PV imaging. No subject had significant (≥70%) PV stenosis. Change in aggregate PV cross-sectional area was less with PFA (−0.9%) than thermal ablation (−12%, posterior probability > 0.999)—primarily driven by the RF sub-cohort (−19.5%) vs. CB sub-cohort (−3.3%). Almost half of all PFA PV diameters did not decrease, but the majority (80%) of RF PVs decreased, regardless of PV anatomic location.

**Conclusion:**

In this first randomized comparison of PFA vs. thermal ablation, PFA resulted in less PV narrowing—thereby underscoring the qualitatively differential and favourable impact of PFA on PV tissue.

## Graphical Abstract

**Graphical Abstract:**
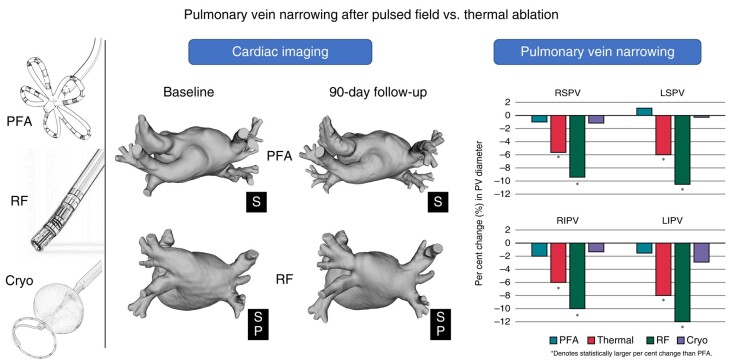

## Introduction

Catheter-based pulmonary vein isolation (PVI) is an effective therapeutic option for patients with atrial fibrillation (AF), and its indications have been rapidly expanding.^1–5^ The conventional energy sources used for PVI include radiofrequency (RF) and cryothermy, which produce lesions by heating or freezing cardiac tissue, respectively. Unfortunately, both RF and cryoablation are associated with several complications including pulmonary vein (PV) stenosis. Narrowing of the PV results from fibrosis of necrotic myocardium, intimal thickening, thrombus formation, endocardial contraction, and proliferation of elastic lamina—all secondary to thermal injury.^6^ Although severe PV stenosis is rare, mild–moderate PV narrowing has been observed in up to 31% of patients with both RF and cryoablation.^7,8^ While the majority of patients with mild–moderate PV narrowing remain asymptomatic, some develop increased PV flow velocity^9^ and, in others, late progression can occur leading to dyspnoea, cough, and haemoptysis.^10^

Pulsed field ablation (PFA) is a largely non-thermal energy approach that involves the use of microsecond-scale, high-voltage electric fields to cause irreversible electroporation and destabilization of cell membranes, a process that culminates in selective cellular necrosis.^11–13^ The acute tissue changes associated with PFA do not exhibit micro-vascular obstruction and intramural haemorrhage seen with thermal ablation.^14^ Moreover, PFA causes less chronic fibrosis than thermal ablation, likely secondary to a specific reparative process leading to preserved tissue compliance,^14^ which may reduce PV narrowing following AF ablation. Until recently, the lack of PV narrowing with PFA was described only in observational studies.^15,16^

The randomized *ADVENT* study demonstrated that PFA was non-inferior to conventional thermal ablation with respect to the primary endpoint of freedom from a composite of initial procedural failure, documented atrial tachyarrhythmia after a 3-month blanking period, anti-arrhythmic drug (AAD) use, cardioversion, and repeat ablation, as well as with respect to device- and procedure-related serious adverse events at 1 year.^13^ The results also demonstrated superiority of PFA to thermal ablation with respect to PV narrowing at 3-month follow-up,^13^ which was secondary endpoint of ADVENT. In this study, we provide an in-depth analysis of this secondary endpoint, and we compare the changes in PV ostial dimensions between the PFA and thermal groups.

## Methods

*ADVENT* was a prospective, multi-centre, randomized, single-blind, non-inferiority pivotal study comparing PFA (pentaspline catheter) with thermal ablation—either force-sensing RF or cryoballoon (CB) ablation for the treatment of paroxysmal AF (NCT04612244).^13,17^ The study was performed in accordance with the US Code of Federal Regulations, Good Clinical Practice, and ethical principles consistent with the Declaration of Helsinki. Institutional Review Board approval was obtained at all investigational sites. Informed written consent was obtained from all trial participants prior to enrolment and randomization.

### Ablation procedure

Patients with symptomatic paroxysmal AF resistant or intolerant to at least one AAD (Classes I–IV) were enrolled.^17^ Patients were randomized 1:1 to PFA or thermal ablation to achieve PVI. Each of the participating centres employed either RF or CB ablation as their thermal control arm. The ablation interventions have been previously described.^17^ Anticoagulation was performed based on standard of care. Sedation or general anaesthesia was used according to institutional protocol. A bolus of heparin was delivered prior to or immediately following trans-septal puncture, with procedural activated clotting times maintained at a minimum of 300 s. Following PVI, the entrance block was confirmed after a 20-min waiting period.

#### Pulsed field ablation

Subjects randomized to PFA underwent PVI using the pentaspline PFA catheter (Farawave, Boston Scientific Inc), deflectable sheath (Faradrive, Boston Scientific Inc), and PFA generator (Farastar, Boston Scientific Inc). Pulsed field ablation applications were performed as previously described,^13^ with a minimum of eight PFA applications delivered to each PV. Each PFA application consists of five packets of pulses delivered over 2.5 s, which were not gated to the QRS complex. These lesions were delivered at 1800, 1900, or 2000 V using the PFA generator. Per protocol, intra-cardiac echocardiography was used during PFA to monitor catheter positioning. Oesophageal management strategies (e.g. temperature monitoring, mechanical deviation, and cooling) during PFA procedures were discouraged. Additional applications were delivered per operator preference if the PV was not isolated.

#### Thermal ablation

Pulmonary vein isolation with thermal ablation was performed with commercially available devices. Oesophageal protection was performed based on institution protocols. Radiofrequency ablation was performed with a conventional saline-irrigated force-sensing RF ablation catheter in conjunction with an electroanatomical mapping system. Radiofrequency applications were delivered (typically 25–50 W) to create a circumferential lesion set to isolate the PVs, either individually or as ipsilateral pairs. During RF ablation, care was taken to ensure that the lesions were antral to avoid PV stenosis. Cryoballoon ablation was performed with a clinically available CB ablation catheter (either 23 or 28 mm, per operator discretion), advanced over a guidewire to the ostium of each PV to deliver cryothermal lesions (typically 2–4 min/lesion). Online monitoring of the PV potentials was optionally used to guide lesion duration. Bonus ablation lesions were delivered per operator discretion.

### Cardiac imaging and pulmonary vein dimensions

Pulmonary vein diameter and aggregate cross-sectional area were obtained at the index procedure using either computed tomography (CT) or magnetic resonance imaging (MRI). At the 3-month follow-up visit, cardiac CT or MRI scanning of the same type as baseline was performed to assess the dimensions of each PV, using the same plane to measure PV area. For subjects with 70% or greater PV diameter reduction, a 12-month follow-up cardiac CT or MRI scan (same type performed at baseline) was scheduled.

**Figure 1:**
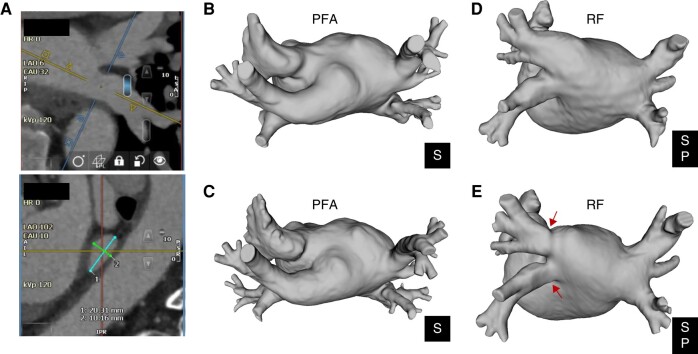
Example cardiac CT measurements showing the short and long diameter measurements (*A*) and images reconstructed in 3D renderings to show pre-procedural and 90-day pulmonary vein dimensions for a patient treated with PFA (*B*, *C*) and thermal (RF) ablation (*D*, *E*). P, posterior; PFA, pulsed field ablation; RF, radiofrequency; S, superior.

All cardiac CT and MRI scans were analysed by a core laboratory. Pulmonary vein diameters were measured by an independent cardiologist, blinded to the treatment arm using Circle 42® (version 5.12.2), a cardiovascular imaging post-processing software for viewing and analysing cardiac CT and MRI images. To evaluate each PV, the centrelines in two orthogonal long-axis planes were determined, and from there, a cross-sectional plane orthogonal to these two planes was defined at the midpoint of the vein between the left atrium and first bifurcation. In this plane, each PV was measured in two orthogonal diameters approximating the longest and shortest diameters (*Figure 1A*). The measured PV diameter was defined as the mean of these two measurements, and the PV cross-sectional area was computed using the formula for area of an ellipse, where the longest and shortest axis measurements of the PV diameter serve as the major and minor axes of the ellipse. The aggregate PV cross-sectional area was the sum of the calculated PV cross-sectional area of each PV ablated at the index procedure.

### Statistical analysis

Continuous variables are reported as mean ± standard deviation. Categorical variables are summarized as count and percentage. Using a Bayesian version of a *t*-test with non-informative prior distributions, the pre-specified, formally tested, secondary safety endpoint compared the change in aggregate PV cross-sectional area from baseline to 3 months between PFA vs. thermal groups, with superiority defined by posterior probability > 0.975. Additional comparisons of changes in PV dimensions across PVs and across modalities were performed using the same methodology^17^ with 95% Bayesian credible intervals (BCIs) for the difference in means derived using uniform priors on (µ, log(σ)) scale and 95% BCIs for the difference in proportions derived using Beta priors with parameters of 0.5 and 0.5.

## Results

### Patients

**Figure 2:**
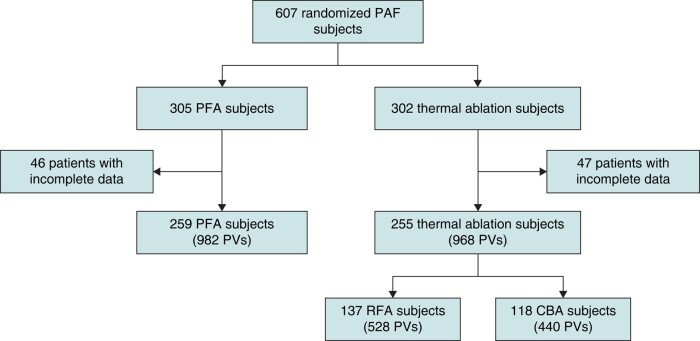
Patient flow chart (only the four main PVs were included in this analysis). CBA, cryoballoon ablation; PAF, paroxsymal atrial fibrillation; PFA, pulsed field ablation; PV, pulmonary vein; RFA, radiofrequency ablation.

Overall, 607 randomized paroxysmal AF patients (305 PFA; 302 thermal ablation) were included in the *ADVENT* trial. Cardiac imaging compliance was 99.7% at baseline and 91.9% at 3-month follow-up. Of the full 607 patient cohort, 93 randomized subjects (15.3%) were not included in the secondary safety endpoint due to missing scan data (46 in the PFA arm and 47 in the thermal arm). Most exclusions were due to missing 3-month follow-up scans (9.2% and 11.3% for PFA and thermal, respectively). Overall, 259 PFA subjects and 255 thermal subjects were included in the analysis. Patient demographics are presented in *Table 1*. There were no clinically meaningful differences at baseline between the groups treated with PFA, RF, or CB ablation. A per-PV accounting using the four main PVs [right superior PV (RSPV), right inferior PV (RIPV), left superior PV (LSPV), and left inferior PV (LIPV)] was also performed and consisted of 982 PVs treated with PFA and 968 PVs treated with thermal ablation (*Figure 2*).

**Table 1:** Patient demographics for patients with compliant baseline and 3-month cardiac imaging for PV dimensional analysis

	PFA	Thermal	Bayesian credible interval	RFA	Bayesian credible interval	CBA	Bayesian credible interval
*N*	259	255		137		118	
Age (yrs)	62.3 ± 8.8	62.8 ± 8.3	(−2.0, 1.0)	62.9 ± 8.1	(−2.4, 1.1)	62.7 ± 8.6	(−2.3, 1.5)
Sex, F (%)	86 (33.2%)	89 (34.9%)	(−9.9, 6.5)	45 (32.8%)	(−9.5, 9.9)	44 (37.3%)	(−14.6, 6.1)
Co-morbidities							
Hypertension	143 (55.2%)	136 (53.3%)	(−6.7, 10.4)	79 (57.7%)	(−12.5, 7.8)	57 (48.3%)	(−3.9, 17.6)
Diabetes	30 (11.6%)	25 (9.8%)	(−3.6, 7.1)	13 (9.5%)	(−4.6, 8.0)	12 (10.2%)	(−5.8, 7.7)
Stroke/TIA	10 (3.9%)	12 (4.7%)	(−4.5, 2.7)	7 (5.1%)	(−6.2, 2.8)	5 (4.2%)	(−5.5, 3.5)
CAD	31 (12.0%)	47 (18.4%)	(−12.6, −0.3)	27 (19.7%)	(−15.8, −0.3)	20 (16.9%)	(−13.2, 2.4)
LVEF (%)	60.3 ± 5.9	59.6 ± 6.0	(−0.3, 1.7)	60.5 ± 5.9	(−1.5, 1.0)	58.5 ± 6.0	(0.5, 3.1)
LA diameter (mm)	38.8 ± 5.8	39.7 ± 6.0	(−1.9, 0.1)	39.3 ± 6.2	(−1.7, 0.8)	40.2 ± 5.8	(−2.6, −0.1)
Days to CT/MRI^a^	102.3 ± 35.4	99.6 ± 28.1	(−2.8, 8.2)	96.0 ± 18.6	(0.9, 11.7)	103.7 ± 35.8	(−9.3, 6.3)

Data are presented as mean ± SD or *N* (%).

95% BCIs calculated for (PFA—thermal) difference.

CAD, coronary artery disease; CBA, cryoballoon ablation; CT, computed tomography; LA, left atrium; LVEF, left ventricular ejection fraction; MRI, magnetic resonance imaging; TIA, transient ischemic attack.

^a^Mean days from procedure to 90-day scan.

### Ablation characteristics

In the 259 patients treated with PFA, an average of 10.0 ± 3.6 applications were performed per PV with the majority of applications (86%) performed at 1.9 kV. For those treated with RF, the average number of applications per PV was 16.2 ± 13.1 at an average power of 39.1 ± 7.2 W, and patients treated with CB ablation received on average 2.1 ± 1.3 freeze applications per PV for an average duration of 161.8 ± 33.1 s.

### Changes in pulmonary vein dimensions

**Figure 3:**
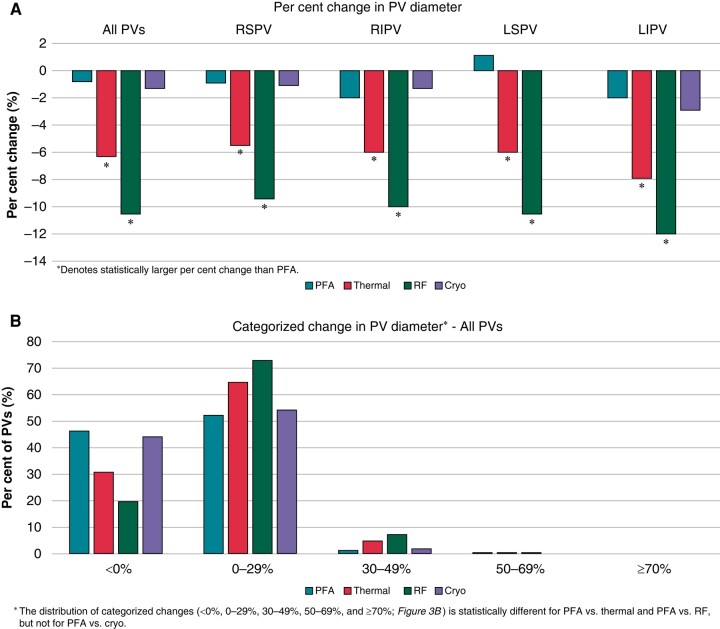
(*A*) Percentage change in pulmonary vein dimensions following PVI by ablation modality. (*B*) Categorization of pulmonary vein diameter changes. PFA, pulsed field ablation; PV, pulmonary vein; RFA, radiofrequency.

**Figure 4:**
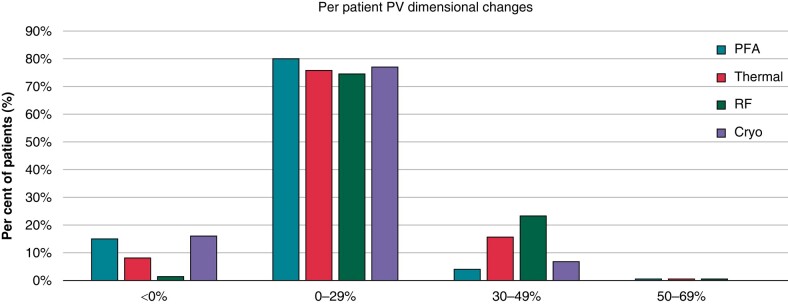
Pulmonary vein diameter changes categorized per patient. PFA, pulsed field ablation; PV, pulmonary vein; RFA, radiofrequency.

There were statistical reductions in the long- and short-axis diameter of the PVs after ablation with thermal energy but not with PFA (*Table 2*). The reduction in diameter with thermal ablation was observed in all PVs: RSPV, RIPV, LSPV, and LIPV (*Table 3*). No subject in either group was found to have significant PV stenosis, defined as a reduction in calculated PV diameter of 70% or greater. In addition, multivariate analysis of the patient demographics did not reveal predictors of PV narrowing. The reduction in diameter with thermal energy occurred predominantly in the RF sub-group (*Figure 3A*). In patients with PV narrowing, the change in PV dimension could be easily seen on post-procedure CT and MR images (*Figure 1*). When categorized by percentage of PVs with any degree of diameter change, only 30% of the PVs in the thermal group showed no change in the PV diameter, while the remaining showed at least some degree of PV diameter reduction. The distribution of categorized changes in all PVs (<0%, 0–29%, 30–49%, 50–69%, and ≥ 70%) is statistically different between PFA and thermal ablation PVs. Further evaluating by thermal modality, the distribution between PFA and RF is statistically different, but not for PFA vs. cryo (*Figure 3B*). The LIPV demonstrated the most narrowing at 12%. When analysed on a per-patient basis, narrowing in at least one PV of <0%, 0–29%, 30–49%, and 50–69% was seen in 15.1%, 80.3%, 4.2%, and 0.4% of PFA patients and 8.2%, 75.7%, 15.7%, and 0.4% of thermal ablation patients (*Figure 4*).

**Table 2:** Pulmonary vein ostial diameters (mm) before and after ablation, by ablation modality

PV ostial dimensions (mm)		Pulsed field ablation				Thermal ablation			
	Axis	*N*	Pre	Post	*Posterior probability*	*N*	Pre	Post	*Posterior probability*
**RSPV**	Long	257	20 ± 3.1	19.6 ± 3.2	0.93	255	19.3 ± 3.1	18.6 ± 3.0	>0.99
	Short		16.6 ± 3.1	16.4 ± 3.1	0.79		16.6 ± 3.1	15.5 ± 2.9	>0.99
**RIPV**	Long	257	18.9 ± 3.2	18.4 ± 3.3	0.96	255	19.0 ± 3.2	17.8 ± 3.3	>0.99
	Short		16.0 ± 3.0	15.5 ± 2.9	0.97		16.0 ± 3.0	14.8 ± 3.1	>0.99
**LSPV**	Long	234	18.5 ± 2.8	18.6 ± 3.0	0.42	229	18.5 ± 3.0	17.4 ± 3.3	>0.99
	Short		13.6 ± 2.6	13.9 ± 2.6	0.21		13.7 ± 2.9	12.7 ± 2.7	>0.99
**LIPV**	Long	234	18.3 ± 2.6	17.9 ± 2.8	0.95	229	18.5 ± 2.3	16.9 ± 2.8	>0.99
	Short		12.2 ± 2.6	12.1 ± 2.6	0.77		12.3 ± 2.8	11.4 ± 2.8	>0.99
**Total**	Long	982	19.0 ± 3.0	18.6 ± 3.1	0.99	968	19.0 ± 3.0	17.7 ± 3.2	>0.99
	Short		14.7 ± 3.3	14.5 ± 3.3	0.89		14.8 ± 3.4	13.7 ± 3.3	>0.99

PV ostial dimensions (mm)		Radiofrequency ablation				Cryoballoon ablation			
	Axis	*N*	Pre	Post	*Posterior probability*	*N*	Pre	Post	*Posterior probability*
**RSPV**	Long	137	20.2 ± 3.1	18.1 ± 2.9	>0.99	118	19.6 ± 3.2	19.1 ± 3.0	0.80
	Short		16.7 ± 3.1	15.0 ± 2.9	>0.99		16.6 ± 3.0	16.1 ± 2.9	0.91
**RIPV**	Long	137	19.3 ± 3.1	17.4 ± 3.1	>0.99	118	18.7 ± 3.2	18.3 ± 3.4	0.81
	Short		16.3 ± 3.0	14.4 ± 2.9	>0.99		15.5 ± 3.0	15.2 ± 3.2	0.78
**LSPV**	Long	127	18.8 ± 3.1	16.9 ± 3.3	>0.99	102	18.1 ± 2.8	18.0 ± 3.1	0.61
	Short		13.9 ± 2.9	12.1 ± 2.8	>0.99		13.5 ± 2.8	13.3 ± 2.5	0.65
**LIPV**	Long	127	18.7 ± 2.3	16.3 ± 2.9	>0.99	102	18.2 ± 2.4	17.6 ± 2.5	0.96
	Short		12.2 ± 3.0	10.9 ± 3.0	>0.99		12.4 ± 2.5	12.0 ± 2.4	0.85
**Total**	Long	528	19.3 ± 3.0	17.2 ± 3.1	>0.99	440	18.7 ± 3.0	18.3 ± 3.1	0.96
	Short		14.9 ± 3.5	13.2 ± 3.4	>0.99		14.6 ± 3.3	14.3 ± 3.2	0.94

Posterior probabilities of smaller PV ostial dimensions post-ablation compared with pre-ablation.

LIPV, left inferior PV; LSPV, left superior PV; PV, pulmonary vein; RIPV, right inferior PV; RSPV, right superior PV.

**Table 3:** Percentage change (%) in pulmonary vein ostial dimensions

Per cent change in ostial dimensions								
	Axis	PFA	Thermal	*Posterior probability*	RFA	*Posterior probability*	CBA	*Posterior probability*
**RSPV**	Long	−1.4 ± 12.0	−5.3 ± 15.7	>0.99	−9.5 ± 13.3	>0.99	−0.3 ± 17.0	0.28
	Short	0.4 ± 18.2	−5.1 ± 18.3	>0.99	−8.4 ± 16.3	>0.99	−1.3 ± 19.8	0.78
**RIPV**	Long	−1.8 ± 14.0	−5.6 ± 15.1	>0.99	−9.3 ± 14.7	>0.99	−1.2 ± 14.4	0.35
	Short	−1.6 ± 17.3	−5.9 ± 18.3	>0.99	−10.3 ± 17.5	>0.99	−0.8 ± 17.9	0.35
**LSPV**	Long	0.7 ± 11.3	−5.0 ± 18.0	>0.99	−9.2 ± 18.7	>0.99	0.2 ± 15.6	0.62
	Short	2.6 ± 15.1	−5.9 ± 20.8	>0.99	−11.3 ± 21.6	>0.99	0.9 ± 17.6	0.80
**LIPV**	Long	−1.9 ± 11.6	−8.3 ± 13.4	>0.99	−12.4 ± 14.5	>0.99	−3.1 ± 9.7	0.85
	Short	0.2 ± 18.8	−6.1 ± 18.7	>0.99	−9.8 ± 19.8	>0.99	−1.5 ± 16.2	0.80
**Total**	Long	−1.1 ± 12.3	−6.0 ± 15.6	>0.99	−10.1 ± 15.4	>0.99	−1.1 ± 14.5	0.50
	Short	0.3 ± 17.4	−5.7 ± 19.0	>0.99	−9.9 ± 18.8	>0.99	−0.7 ± 17.9	0.85

Posterior probability of superiority (i.e. less reduction in PV ostial dimension) of PFA to thermal/RF/cryo.

CBA, cryoballoon ablation; LIPV, left inferior PV; LSPV, left superior PV; PFA, pulsed field ablation; PV, pulmonary vein; RFA, radiofrequency ablation; RIPV, right inferior PV; RSPV, right superior PV.

### ADVENT secondary safety endpoint: pulmonary vein cross-sectional area

**Figure 5:**
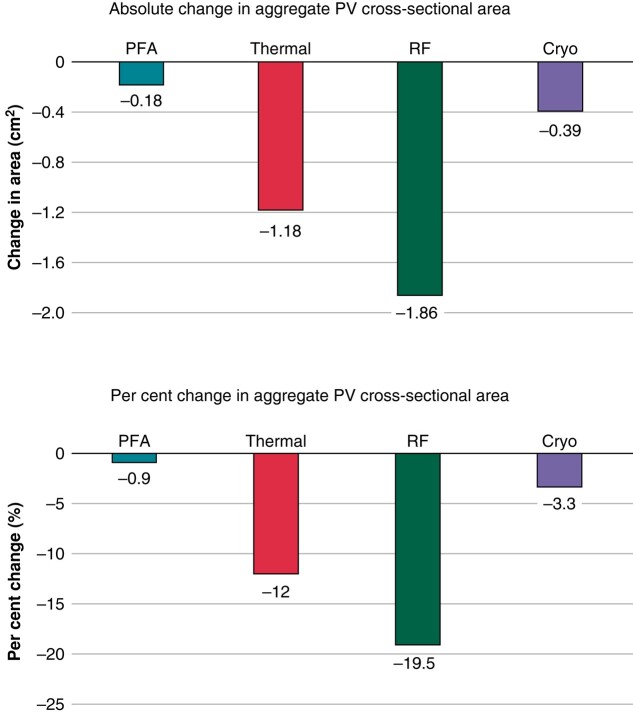
Absolute and per cent change in aggregate pulmonary vein cross-sectional area by ablation modality from baseline to 3-month follow-up. PFA, pulsed field ablation; PV, pulmonary vein; RFA, radiofrequency.

The change in PV diameter translated into a reduction in the PV cross-sectional area, and the secondary safety endpoint met its success criterion for superiority of PFA to thermal ablation. The change in aggregate PV cross-sectional area from baseline to Day 90 was less in PFA subjects (−0.18 cm^2^ or 0.9%) than in thermal subjects (−1.18 cm^2^ or 12.0%) (posterior probability of superiority > 0.999). This reduction in PV cross-sectional area was largely driven by the RF ablation sub-group (−1.86 cm^2^ or 19.5%; *Figure 5*)

## Discussion

This study reports a randomized analysis of PV narrowing between PFA and thermal ablation. The pre-specified secondary safety end point of ADVENT was met, demonstrating that PFA results in significantly less PV narrowing than thermal ablation.

### Incidence of pulmonary vein narrowing during pulmonary vein isolation

Pulmonary vein isolation using thermal ablation continues to be associated with PV narrowing despite many years of experience and advances in imaging and technologies for ablation. Severe PV stenosis is rare but lesser degrees of narrowing are more common. Teunissen *et al*.^7^ reported that mild PV stenosis can occur in 31.4% of patients undergoing RF ablation, which is similar to the 30.6% rate with CB ablation reported by Narui *et al*.^8^ Indeed, the rate of PV narrowing in ADVENT with thermal ablation is similar to previously published data using RF. The PV with the most narrowing from RF was the LIPV, which is likely related to the ablation catheter impinging into the PV ostium with the ablation at the anterior/inferior aspect of the PV, where acute angulation may make catheter control proximally more challenging. The lack of change in PV calibre with PFA confirms the findings of prior observational studies that reported the absence of PV narrowing with PFA.^15,16^

### Mechanism of pulmonary vein narrowing

The mechanism of action of thermal ablation involves temperature changes that can lead to micro-vascular obstruction and intramural haemorrhage, which in turn causes fibrosis. Dense fibrosis and contraction of the scar can lead to PV narrowing when energy is applied within or at the orifice of the PVs.^18^ In contrast, the therapeutic effect of PFA is non-thermal and instead employs high-voltage electric fields to irreversibly electroporate cells. A comparative histopathological pre-clinical study showed significant differences between PFA and RF lesions: first, remodelling of the cardiac wall into fibrotic tissue after ablation is more homogeneous with PFA than thermal ablation; second, PFA does not seem to cause arteriolar remodelling, and blood vessels within PFA lesions are largely spared; and third, intimal and medial hyperplasia and thrombosis were seen more frequently with PFA than RF ablation.^19^ Another clinical study using MRI of chronic ablation lesions showed that PFA causes less chronic fibrosis than thermal ablation, which was attributed to a secondary reparative process leading to preserved tissue compliance.^14^

### Clinical implications

Most patients with mild-moderate PV narrowing remain asymptomatic, but studies show that some can develop clinical signs and symptoms of exercise intolerance. A study utilizing trans-oesophageal echocardiography reported that patients with moderate stenosis can develop dyspnoea and increase PV flow velocity.^9^ Further, by Poiseuille’s law, changes in vessel radius translate to significant changes in vascular resistance, such that even seemingly small decreases in PV dimensions can lead to a meaningful increase in vascular resistance.^20^ Limiting these effects through the use of PFA may have important implications in patients with heart failure or pulmonary hypertension.^21^

Another important consideration is that PV narrowing can be progressive.^22^ Mild–moderate PV narrowing at 3 months after ablation could progress into a more severe narrowing and is the strongest predictor for severe PV stenosis after reablation.^23^ Late progression may lead to symptoms such as dyspnoea, cough, and haemoptysis long after ablation has been previously described.^10^ As a result, asymptomatic mild–moderate narrowing of the PV after ablation should not be considered non-consequential, and its absence with PFA provides a significant advantage for this ablation energy source.

### Limitations

There are several limitations of this sub-study. Despite 607 patients included in the ADVENT study and 514 patients with complete PV imaging data, there were no cases of clinical PV stenosis identified and the data presented here are on a subset of the full patient cohort (259 PFA and 255 thermal ablation). On the other hand, the thermal procedures in ADVENT were performed by expert operators; PV stenosis invariably does occur in standard clinical practice including the broad base of operators with variable clinical experience and expertise. Combined with the qualitatively differential effects of PFA on PV calibre (not even a small degree of PV ‘waisting’, unlike with thermal ablation), these sub-critical reductions in PV calibre with thermal ablation have significant implications. One additional limitation is that there was no protocol-mandated workflow for the thermal arm—RF or CB ablation—which might have influenced the degree of PV narrowing. In addition, there is limited follow-up with a single time point for re-evaluating PV dimensions at 3-month follow-up after the procedure, and this may warrant further investigation at longer follow-up time points, like what was used in a prior study.^24^

## Conclusion

In this randomized study of PFA vs. thermal ablation for treating paroxysmal AF, PFA resulted in statistically less PV narrowing measured as aggregate cross-sectional area. These data underscore the qualitatively differential and favourable impact of the non-thermal energy modality, PFA, on PV tissue.

## Data Availability

The data from this clinical trial may be made available to other researchers in accordance with Boston Scientific's Data Sharing Policy (http://www.bostonscientific.com/en-US/data-sharing-requests.html).
